# Editorial: Strategies to overcome metabolic syndrome and related diseases

**DOI:** 10.3389/fphys.2024.1501333

**Published:** 2024-10-02

**Authors:** Lin Zhu, Curtis C. Hughey, Marica Bakovic, William J. Massey

**Affiliations:** ^1^ Division of Endocrinology, Metabolism, and Diabetes, Department of Medicine, Vanderbilt University Medical Center, Nashville, TN, United States; ^2^ Division of Molecular Medicine, Department of Medicine, University of Minnesota Twin Cities, Minneapolis, MN, United States; ^3^ Department of Human Health and Nutrition Sciences, College of Biological Science, University of Guelph, Guelph, ON, Canada; ^4^ Department of Inflammation and Immunity, Lerner Research Institute, Cleveland Clinic, Cleveland, OH, United States; ^5^ Center for Microbiome and Human Health, Lerner Research Institute, Cleveland Clinic, Cleveland, OH, United States

**Keywords:** metabolic syndrome (MetS), cardiovascular disease, hypertension, obesity, lipodystrophy, physical activities, adipose tissues

Metabolic syndrome (MetS) is a cluster of conditions that include increased blood pressure, elevated blood glucose, central obesity, hyperlipidemia, and low HDL cholesterol content (Liang et al., 2021). The diagnosis of MetS requires the presence of three or more of those metabolic conditions. MetS-related diseases include type 2 diabetes, cardiovascular disease, cognitive dysfunction, stroke, and cancers. According to the United States National Health and Nutrition Examination Survey (NHANES) 2011–2018, MetS prevalence increased from 37.6% in 2011–2012 to 41.8% in 2017–2018 among adults aged 20 years or older (Liang et al., 2021). This rising prevalence is concerning because MetS and related diseases would decrease individual life quality and burden the healthcare system (Grundy, 2008). Furthermore, current therapeutic interventions, including pharmaceutical agents and lifestyle modification, mitigate but do not abolish all components of MetS and its co-morbidities. This limitation highlights the importance of improving our understanding of the underlying mechanisms and targets for improving MetS. Welcome to the present Research Topic, which assembles 15 articles, including five review papers, covering nutritional interventions in animal models, clinical trials for management of dyslipidemia, epidemiologic studies regarding environmental factors, and the current understanding of molecular pathways.

It has been reported that up to 87% of individuals with MetS have dyslipidemia (Toh and Lee, 2020). Given this, research into the key factors regulating lipid homeostasis in MetS and its comorbidities has received a vast amount of attention. In this Research Topic, Nauli et al. Explored the effects of sex hormones on the size of intestinal lipoproteins that carry dietary lipids to the periphery (Nauli et al.). Compared to women, men are more prone to central obesity, one of the five conditions that comprise MetS. Using the conscious lymph fistula mouse model, the authors discovered that male mice produced larger intestinal lipoproteins than female mice when intraduodenally infused with lipid emulsion (Nauli et al.). Larger lipoproteins may contribute to central obesity by facilitating increased fat uptake in visceral abdominal adipose tissue. Further investigations leveraging the Caco-2 cell model showed that testosterone significantly increased the size of lipoproteins in a dose-dependent manner (Nauli et al.). Regarding whole-body cholesterol balance, Banerjee et al. reported that, unlike the Aster-B protein, which plays an essential role in cholesterol transport and downstream esterification, the non-vasicular Aster-C protein (encoded by Gramd1c gene) played a minor role in whole-body cholesterol balance in a sophisticated study including divergent dietary cholesterol and Gramd1c knockout mouse model (Banerjee et al.). The gut hormone glucagon-like peptide-2 (GLP-2) has been shown to play pleiotropic roles in regulating lipid handling in the intestine, which is important for maintaining energy homeostasis and cardiometabolic health. In the review by Mukherjee and Xiao, the authors elucidated the mechanisms of GLP-2 in regulating post-prandial lipid absorption and post-absorptive release of intestinally stored lipids (Mukherjee and Xiao). While further summarizing the role of GLP-2 in metabolic disorders, the authors discussed the opportunities to promote health benefits beyond its current clinical use for treating short-bowel syndrome by manipulating GLP-2 mediated pathways (Mukherjee and Xiao).

MetS is closely linked to dysregulated energy homeostasis. Engaging in daily physical activities has become the first choice in clinics for managing MetS. Physical activities are well known for body weight management by promoting energy expenditure. However, the impact of physical activities on CVD-related HDL function has yet to be further studied. In a human study including healthy lean, obese, and type 2 diabetic subjects, Zhu et al. demonstrated that a short-term high-intensity interval training (HIIT) program improved HDL function depending on metabolic contexts, correlating with improvements in blood lipid profile (Zhu et al.). This study showed that triacylglycerol content in HDL particles may negatively affect the anti-atherogenic function of HDL (Zhu et al.). Furthermore, Sadler et al. quantified the impact of protonophore treatment on whole-body energetics in mice housed at 30°C, a thermoneutral housing that may mask the effects of anti-obesity strategies on energy expenditure (Sadler et al.). They observed that mice housed at 30°C showed reduced basal energy expenditure compared to 24°C controls, and protonophore treatment markedly increased energy expenditure, resulting in reduced adiposity in mice housed at 30°C (Sadler et al.). Nicotinamide N-methyltransferase (NNMT) is involved in energy expenditure by lowering NAD^+^ content via catalyzing the methylation of nicotinamide. Sun et al. reviewed the current understanding of the role of NNMT in MetS (Sun et al.). Single nucleotide variants in the NNMT gene are significantly correlated with disturbances in energy metabolism; elevated NNMT gene expression is notably observed in the liver and white adipose tissues of obese individuals (Sun et al.). In animal models, knockdown NNMT expression with RNAi strategies or small molecule inhibitors improved MetS-related diseases (Sun et al.).

Lipodystrophy syndromes are rare diseases, and delays in diagnosis may predispose to the development of severe metabolic complications and end-organ damage. A rapid action plan was developed by Fourman et al. using insights gathered through a series of advisory meetings with clinical experts from multiple countries (Fourman et al.). The Rapid Action Plan includes clinical and family history, physical exams and laboratory criteria, diagnostic tools of imaging and genetic testing, as well as guidelines for the syndrome treatment and management (Fourman et al.). Along with a commentary article by Massey and Zhu, discussions in the Rapid Action Plan highlighted the significant role of adipose tissues in MetS (Fourman et al.; Massey and Zhu). Another clinically relevant research article in this Research Topic is by Bukara-Radujkovic et al. who demonstrated the role of glycemia risk index in managing blood glucose in routine clinical practice by exploring the correlation of traditional parameters, such as HbA1c, and novel parameters, including glycemia risk index and time-in-range, in pediatric patients with type 1 diabetes (Bukara-Radujkovic et al.).

Additionally, Li et al. highlighted the significant role of astrocytes in bridging MetS and cognitive dysfunction (Li et al.). Systemic inflammation and endocrine disruption in MetS may drive neurodegeneration mediated by astrocytes, which sense and integrate metabolic signals with neurological function (Li et al.). In this review, Li and co-authors summarized the alterations in astrocyte phenotypic characteristics in MetS, which could possibly serve as a diagnostic marker or even a therapeutic target for MetS-associated cognitive dysfunction (Li et al.). Furthermore, El-Deen et al. explored the impact of co-administration of apricot kernels and caffeine on MetS in an animal model of diabetes, and Yi et al. showed that acetaminophen administration may improve outcomes in critically ill patients with gout and hypertension (Yi et al.). By analyzing data from the NHANES 2013–2018 survey cycle, Chen et al. observed a negative association between chloroform and type 2 diabetes in older adults in the United States (Chen et al.).

Moreover, Shi reviewed a potential therapeutic target, while Lu et al. reviewed a novel strategy for MetS and related diseases in this Research Topic. Salt-inducible kinases (SIKs) are serine/threonine kinases of the adenosine monophosphate-activated protein kinase family (Shi). Shi summarized the diverse roles of SIKs in sodium sensing and salt intake, vascular remodeling, pulmonary arterial hypertension, cardiac hypertrophy and ischemia, inflammation, fibrosis, and MetS (Shi). Interestingly, SIKs are broadly expressed in relevant metabolic tissues, such as adipose tissues and the liver, and play essential roles in mediating insulin action, lipid metabolism, and energy expenditure (Shi). Pharmaceutical inhibition of SIK activity has shown therapeutic potential in various disease models, including inflammatory and fibrotic diseases (Shi). Genetic variants in SIK genes have also been shown to be linked with alterations in blood lipid panels, however, the potential clinical relevance of SIK for dyslipidemia is yet to be studied further (Shi). Regarding the natural supplements for treating MetS and related diseases, Lu et al. reviewed the role of naringin in treating atherosclerosis (Lu et al.). Naringin is a flavonoid abundantly found in grapefruit and tomatoes. In the liver, naringin can be converted into naringenin by naringinase, which process may be involved in glycemic control (Lu et al.). Lu et al. summarized the anti-atherogenic effects of naringin in lowering blood pressure, improving dyslipidemia, protecting endothelium, and inhibiting vascular smooth muscle cell proliferation and migration (Lu et al.). However, the underlying mechanism is not yet conclusive.

The editor team of this Research Topic would like to thank all the authors, reviewers, and readers who have contributed to this Research Topic. With all your support, we hope this Research Topic may add a piece of the puzzle to the whole picture of MetS while providing a beam of light for future studies in this research field.
